# A feasibility study of 5G positioning with current cellular network deployment

**DOI:** 10.1038/s41598-023-42426-1

**Published:** 2023-09-15

**Authors:** Bernardo Camajori Tedeschini, Mattia Brambilla, Lorenzo Italiano, Simone Reggiani, Davide Vaccarono, Marianna Alghisi, Lorenzo Benvenuto, Alessandro Goia, Eugenio Realini, Florin Grec, Monica Nicoli

**Affiliations:** 1 Dipartimento di Elettronica, Informazione e Bioingegneria (DEIB), Politecnico di Milano, 20133 Milan, Italy; 2 Network Engineering, NTT DATA, 20143 Milan, Italy; 3 Network Strategy and Engineering, Vodafone Italia, 10155 Ivrea, Italy; 4 Dipartimento di Ingegneria Civile e Ambientale (DICA), Politecnico di Milano, 20133 Milan, Italy; 5 algoWatt S.p.A., 16149 Genoa, Italy; 6 Geomatics Research & Development srl (GReD), 22074 Lomazzo, Italy; 7 European Space Agency (ESA), 2201 AZ Noordwijk, The Netherlands; 8 Dipartimento di Ingegneria Gestionale (DIG), Politecnico di Milano, 20133 Milan, Italy

**Keywords:** Electrical and electronic engineering, Computer science, Information technology

## Abstract

This research examines the feasibility of using synchronization signals broadcasted by currently deployed fifth generation (5G) cellular networks to determine the position of a static receiver. The main focus lies on the analysis of synchronization among the base stations of a real 5G network in Milan, Italy, as this has a major impact on the accuracy of localization based on time of arrival measurements. Understanding such properties, indeed, is fundamental to characterize the clock drifts and implement compensation strategies as well as to identify the direct communication beam. The paper shows how the clock errors, i.e., inaccurate synchronization, among 5G base stations exhibit a significant bias, which is detrimental for precise cellular positioning. By compensating the synchronization errors of devices’ clocks, we demonstrate that it is in principle possible to localize a static user with an accuracy of approximately 8–10 m in non-obstructed visibility conditions, for urban and rural scenarios, using the deployed 5G network operating at 3.68 GHz and relying on broadcast signals as defined by 5G Release 15 standard. This work has been funded by the European Space Agency (ESA) Navigation Innovation and Support Program (NAVISP) Element 2 pillar which aims at improving the competitiveness of the industry of the participating States in the global Positioning, Navigation and Timing (PNT) market.

## Introduction

Cellular positioning is a standardized feature of communication protocols since the Third Generation Partnership Project (3GPP) fifth generation (5G) Release 15^1^, where Location Services (LCSs) have been introduced for regulatory use-cases such as lawful interceptions and emergency calls^2, 3^. In 3GPP Release 15, the support for commercial and roaming LCS capabilities is not provided. Moreover, Release 15 does not introduce any novel positioning technique aside from Cell-ID placement, thus reusing the positioning techniques available in the Long Term Evolution (LTE) protocol^4^. It is with 3GPP 5G Release 16^5^ that the supports for both mobile-originated location requests (MO-LR), i.e., initiated by the client, and mobile-terminated location requests (MT-LR), i.e., started by the network, are firstly introduced in a cellular telecommunication standard. This has been achieved by introducing dedicated positioning signals, referred to as Positioning Reference Signals (PRSs)^6^, which enhance the cellular positioning technology by improving the correlation properties of LTE PRS. The distinct and innovative features of PRSs include improved correlation properties with respect to any other 5G signal and improved hearability thanks to the concept of muting^7^. Thereby, such cellular communication standard has the most advanced design of the physical layer to specifically address positioning applications.

The need for an accurate, reliable and secure cellular positioning has emerged in a number of 5G and beyond application verticals^8–15^, such as geolocation, geomarketing, augmented reality, asset monitoring, social media, and more^16^. Moreover, the information on the position of the User Equipment (UE) or network apparata is also strategic for location-aware communications, such as in device-to-device communications^17^, vehicular communications^18–22^, or in new paradigms of wireless communication using reconfigurable intelligent surfaces^23^. The potential impact of cellular technology in delivering breakthrough position-related services embracing new market applications is foreseen by the 3GPP standard documentation, however, at present, a main issue is related to hardware limitation. Specifically, cellular positioning capabilities are strongly dependent on precise network synchronization. If the local clocks of cellular base stations are not accurately synchronized to a common network timing reference, a bias error will be present in measuring time-related parameters, thus impairing precise positioning in Time of Arrival (ToA) and Time Difference of Arrival (TDoA) methods. It is worth noting that this issue does not affect Round Trip Time (RTT) methods, in which range errors are mainly due to the propagation delays and clock drifts accumulated during the RTT.

The synchronization problem has been widely investigated in the literature^24–27^, but it still remains one of the biggest sources of error for precise positioning in cellular networks. For 5G communications, uplink and downlink transmissions between base stations and the UEs are scheduled in temporal slots, thus synchronization among the clocks is required to have a univocal reference of timing. Given the unfeasibility of providing atomic clocks at the UE side synchronized with Navigation Satellite System (GNSS), a Precision Time Protocol version 2 (PTPv2)^28^ was created to meet the synchronization requirements. The PTPv2 protocol behaves according to a primary-and-secondary clock paradigm. The primary reference clock is deployed in the backhaul of the cellular network and provides timestamps to the secondary clock which, on the contrary, is placed at the TRPs (i.e., at the cellular base stations). Then, the secondary clock adjusts its local clock to keep precise time alignment with the primary clock. Although the PTPv2 protocol attracted significant interest for its capabilities of jamming resistance and indoor deployment, actual implementations could only achieve a clock synchronization that is accurate up to $\pm 1.5$ µs, as recommended by the International Telecommunication Union (ITU)^29^. Converting such time error into the distance, an error of up to $\pm 450$ m is introduced, confirming that the cellular network has never been conceived for accurate positioning purposes so far. Therefore, the clock synchronization issue emerges as a key limiting factor for the roll-out of precise 5G LCSs. A definite solution has yet to come, but a possible option to mitigate the problem could be including more precise oscillators such as the Temperature-Controlled Crystal Oscillators (TCXOs), with accuracy of approximately $\pm 100$ ns, or the Oven-Controlled Crystal Oscillators (OCXOs), with accuracy of $\pm 50$ ns^30^. Moreover, additional solutions based on the already available 5G Integrated Access and Backhaul (IAB)^31^ could calculate the synchronization errors across TRPs, store them in a database and share them with the UE as assistance information.

Since 3GPP Release 16 positioning features are foreseen to be operational not earlier than late 2023, and PRSs are not available in the current release, we here assess the positioning capability of current 5G networks using the SSBs^32^. SSBs represent a special category of 5G signals that a TRP periodically broadcasts over spatial communication beams, enabling a UE to establish a cellular connection. The details of the initial access phase are here omitted; the reader can refer to^33^.

**Figure 1:**
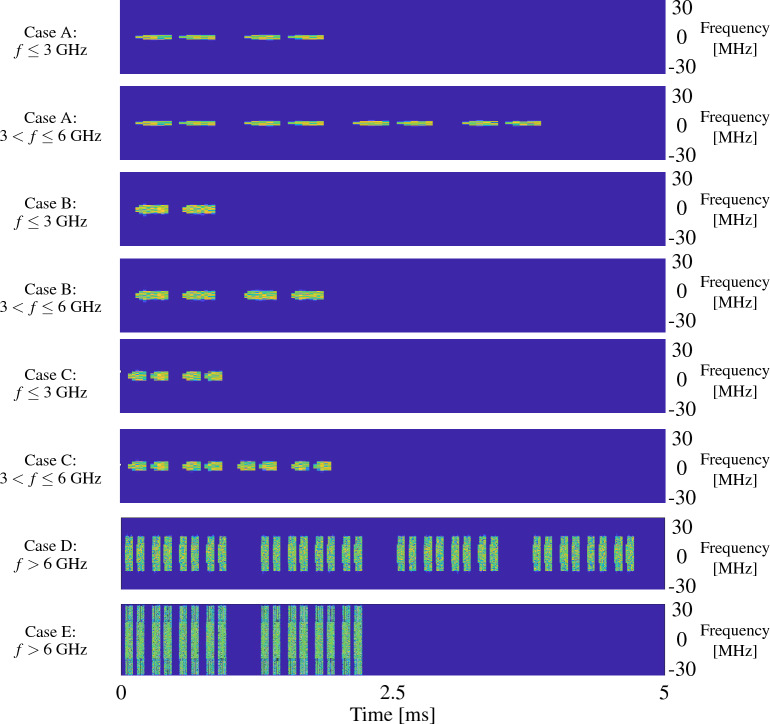
SSB patterns as described by 3GPP Release 15 standard^1^.

Recommended periodicity of SSBs is of 20 ms, although 3GPP standard foresees also other periodicities, i.e., 5 ms, 10 ms, 40 ms, 80 ms and 160 ms^34^. Different time-domain patterns for SSB transmission exist, differing according to the signal frequency *f* (distinguishing among frequencies lower than 3 GHz, between 3 GHz and 6 GHz, and higher than 6 GHz) and Sub-Carrier Spacing (SCS). The patterns are referred to as Case A, B, C, D, and E^35^, as detailed in Table 1. Each pattern is also characterized by the number of consecutive SSBs ($N_{\text {SSB}}$) that are sent in a burst. Intuitively, a pattern specifies how many spatial directional beams a TRP uses to enable its discovery by the UEs. Figure 1 reports all the possible SSB patterns highlighting that a higher number of beams is used for high frequencies (e.g., millimeter waves). Higher values of $N_{\text {SSB}}$ indicate a TRP able to densely scan the spatial domain through more directional beams.

**Table 1:** SSB resource allocation.

SCS	Starting OFDM symbol	Frequency	Frequency	Frequency
		$f \le 3$ GHz	$3 < f \le 6$ GHz	$f > 6$ GHz
Case A: 15 kHz	$\{2, 8\}$ + 14 *n*	$n = 0,1$ $(N_{\text {SSB}}=4)$	$n = 0,1,2,3$ $(N_{\text {SSB}}=8)$	NA
Case B: 30 kHz	$\{4, 8, 16, 20\}$ + 28 *n*	$n = 0$ $(N_{\text {SSB}}=4)$	$n = 0,1$ $(N_{\text {SSB}}=8)$	NA
Case C: 30 kHz	$\{2, 8\}$ + 14 *n*	$n = 0,1$ $(N_{\text {SSB}}=4)$	$n = 0,1,2,3$ $(N_{\text {SSB}}=8)$	NA
Case D: 120 kHz	$\{4,8,16,20\}$ + 28 *n*	NA	NA	$n = \{i\}_{i=0}^{18}$ $(N_{\text {SSB}}=64)$
Case E: 240 kHz	$\{8,12,16,20,32,36,40,44\}$ + 56 *n*	NA	NA	$n = \{i\}_{i=0}^{8}$ $(N_{\text {SSB}}=64)$

In this paper, we measure the ToA of SSB in real urban and rural scenarios with the final aim of assessing the impact of the synchronization error on 5G cellular positioning with the current 5G network deployment. This is done by knowing the periodicity of the SSB pattern and the exact position of the TRPs. The main scientific contribution relies upon a thorough study of the synchronization error (or, equivalently, range bias) and its impact on the positioning accuracy, analyzing urban and rural scenarios with TRPs in visibility and non-visibility conditions. We analyze the decorrelation period and the time drifts of the internal clocks at the base stations. Finally, we investigate the positioning capabilities of the real 5G system assessing both raw and synchronization-compensated ranging measurements.

The paper is organized as follows: Section Results reports the main outcome of the measurement campaigns, i.e., the range bias distributions, the clock decorrelation period and the positioning performances. We further examine the reported analysis in Section Discussion, where we derive conclusions and suggest future research directions. Lastly, in Section Methods, we provide information about the data gathering and data filtering processes, together with the main adopted performance metrics and positioning algorithms.

**Figure 2:**
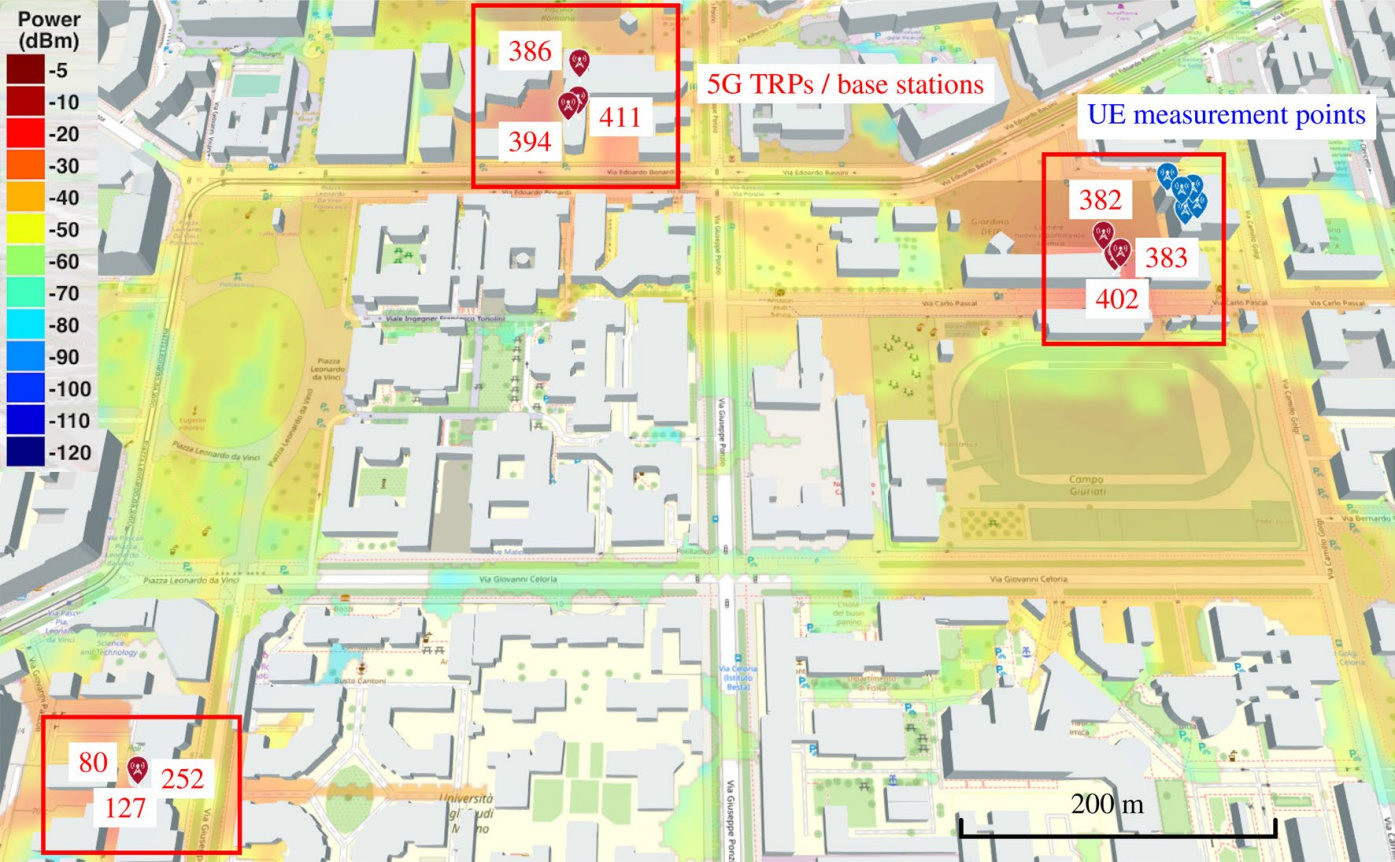
Representation of the urban scenario selected for the experiments and composed of three 5G sites (red squares), each with 3 TRPs (red icons) with indicated PCI identifiers, and 5 UE measurement points (blue icons). The power map is computed with MATLAB ray-tracing software at ground level.

**Figure 3:**
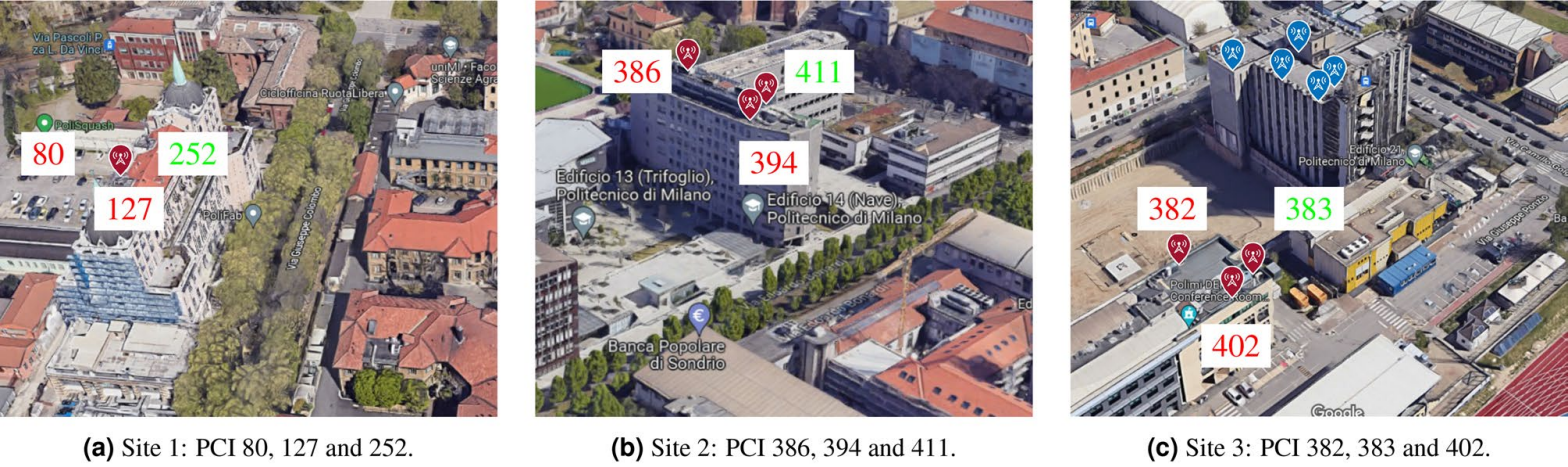
Three sites of the urban scenario in Fig. 2, with related TRPs. For each TRP, the color of PCI number indicates the visibility condition: green color is for LOS, red color is for NLOS.

## Results

We investigate the cellular positioning performance and the impact of synchronization in the Vodafone 5G network deployed in Milan, Italy, operating in the frequency band n78, with carrier frequency of 3.68 GHz. The aim is to assess the network readiness to accommodate precise positioning services. The experiments conducted in this research measure 5G signals as defined by 3GPP Release 15 standard, which does not include PRSs, although the considered Vodafone 5G network is compliant with 3GPP Release 16. The deployment has not undergone any modification or alteration to permit our experiments; we used a Rohde & Schwarz (R&S) TSMA6 scanner to passively receive 5G signals as described in Section Methods. The scanner was placed on top of the Building 21 of the campus Leonardo of Politecnico di Milano such that visibility conditions with at least three TRPs were guaranteed. We remark that the bandwidth of SSB signals is much narrower than the one foreseen for PRSs. The analyses consider a frequency occupancy of 30 kHz for each subcarrier, leading to an overall occupied bandwidth of 7.2 MHz, as SSB pattern C for 5G numerology 1 with periodicity 20 ms is used (20 resource blocks of 12 subcarriers each). The results reported in this section reveal that, at present, the TRPs are subject to a synchronization error that prevents precise positioning (we measure a mean error of 230 ns, which corresponds to a ranging error of 70 m). Clearly, such error needs to be estimated, tracked and compensated by dedicated location measurement units or algorithms to roll out precise 5G positioning services. Nevertheless, the analysis shows that, by clock bias tracking and compensation, in current deployment of 5G networks (Release 15) it would possible to localize a static UE with an error of $8-10$ m as reported in Section Positioning performance.

### Clock offset statistics

We first analyze the statistics of the synchronization errors among the TRPs, under conditions of non-obstructed visibility (i.e., Line-Of-Sight (LOS)) and obstructed visibility (Non-LOS) between a TRP and the UE. The synchronization bias is measured by placing the network scanner in a set of known locations in LOS conditions and computing the error of the range estimates as described in Section Methods.

We consider the urban geographical area in Fig. 2, which reports the nine TRPs under examination (red markers), clustered in three 5G sites (highlighted in Fig. 3) and unequivocally identified by their Physical Cell IDs (PCIs), along with the five measurement points (blue markers) where we put the 5G scanner receiving the SSBs. Note that the figure has been obtained with MATLAB ray-tracing software and it is here reported for contextualization and visualization purposes, providing insights on the 5G layout: the experiments described hereafter do not consider simulations but real measurements. From geometrical analyses through ray-tracing, the TRPs in LOS condition are the ones with PCI number 252, 383, and 411. On the contrary the TRPs with obstructed visibility have PCI numbers 80, 127, 386, 394, 382, 402. Regarding the synchronization aspects, it is obtained by either legacy or Synchronous Ethernet/1588v2 (SFP) via GNSS. Specifically, in our experiments, TRPs with PCIs 80-127-252-382-383-402 are synchronized with PTPv2, while TRPs with PCIs 386-394-411 use SFP via GNSS technology.

**Figure 4:**
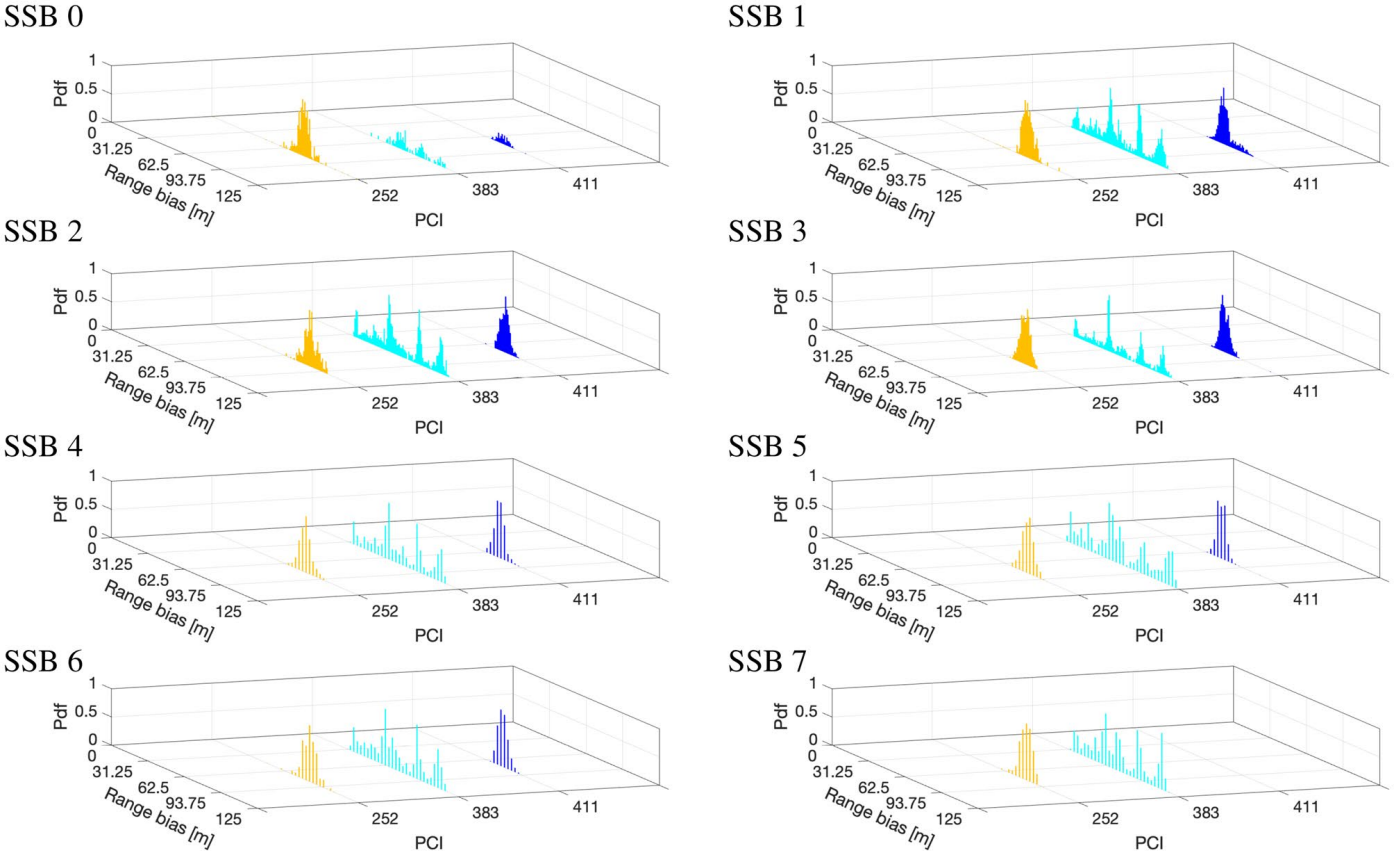
Distribution of the synchronization error in meters, i.e., range bias, for the TRPs in visibility (identified by their PCIs) for each of the 8 SSB beams.

**Figure 5:**
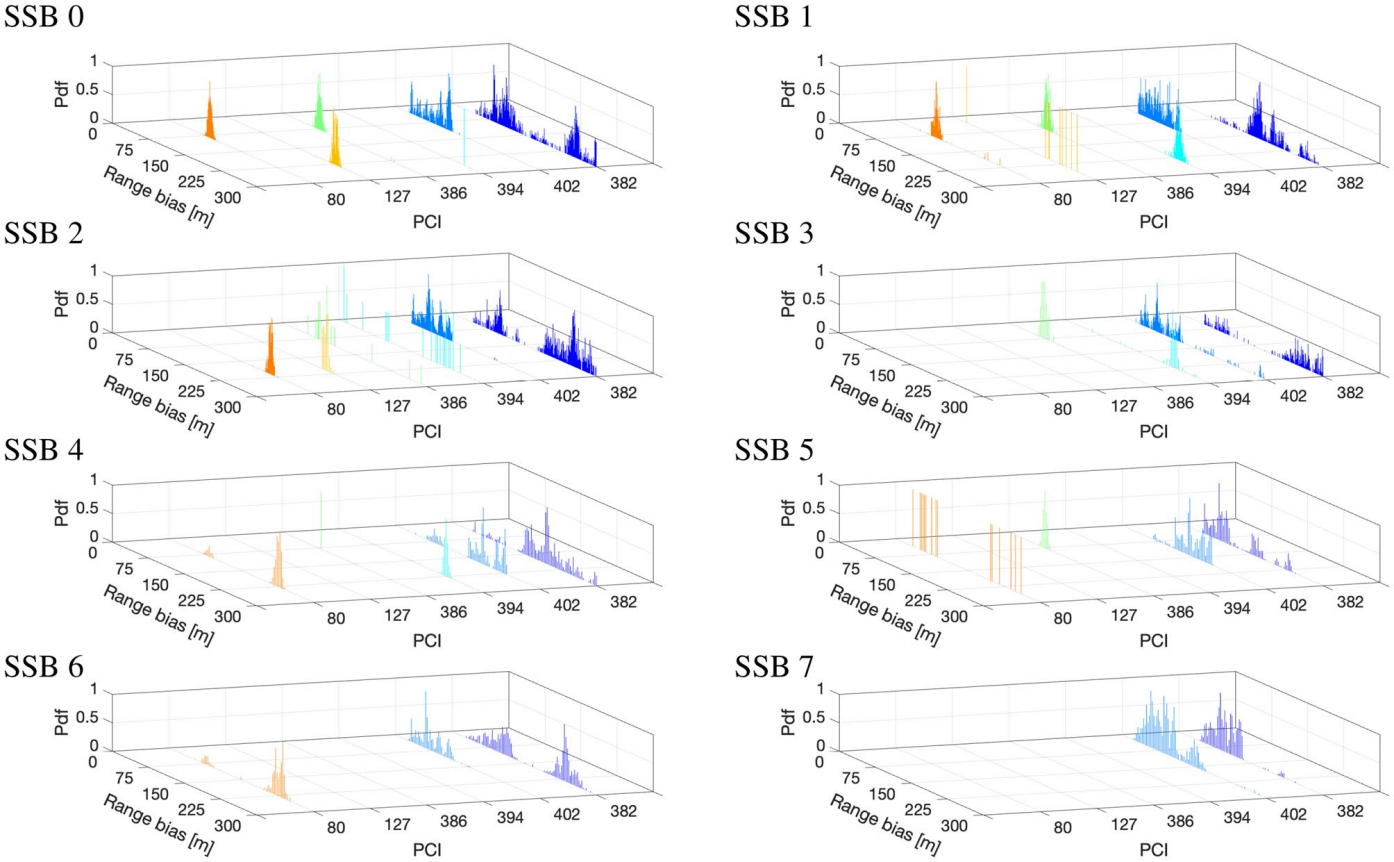
Distribution of the synchronization error in meters, i.e., range bias, for the TRP in non-visibility (identified by their PCIs) for each of the 8 SSB beams.

The analysis of the clock error is reported in Fig. 4 in terms of the histogram of the range bias, i.e., the synchronization error converted in meters, for each of the eight SSB beams of TRPs in visibility. The measured distribution reports a twofold behavior: for PCIs 252 and 411 the range bias distribution is mono-modal (note the presence of one clear peak), while for PCI 383 it presents a wider dispersion without a clearly defined shape. Considering that the visibility condition was well-respected, the mono-modal distribution for PCIs 252 and 411 is somehow understandable as the LOS distribution of the time of flight error is little affected by multipath components. However, we remark that the range error has a mean value (i.e., bias) of 73 m and 79 m for PCIs 411 and 252, respectively. As discussed later in Section Positioning performance, compensation of such bias is a key operation to unlock precise positioning services in 5G networks. Moving to the analysis of the TRP with PCI 383, we notice a completely different distribution. Given that the scanner simultaneously measured the 5G signals received from all TRPs, we attribute such an anomalous behavior to the TRP with PCI 383 itself. In this particular case, TRP with PCIs 383 adopted the PTPv2 standard which provides a high-quality frequency synchronization irrespective of the network load through physical layer (Ethernet interfaces). This protocol can provide a timing accuracy of at least $\pm 100$ ns (i.e., $\pm 30$ m), which can be improved with more precise local oscillators. This aspect is further investigated in Section Clock offset coherence time.

The analysis for non-visibility conditions is reported in Fig. 5. Compared to the LOS case in Fig. 4, the major difference is in the shape of the distribution, which manifests different peaks mostly likely generated by the multipath phenomenon. Due to the NLOS, in fact, the observed range error is generated not only by the clock error, but also by the multipath contributions. Moreover, wider supports, large mean errors (the overall mean for non-visibility TRPs is of 150 m), and absence of received signal is also experienced. Due to the higher emitted power of TRPs, i.e., around $10-20$ W, the LOS contributions are likely to be present, but hard to isolate. A joint tracking over time of the range biases that accounts for the different visibility conditions is advisable^36^.

**Figure 6:**
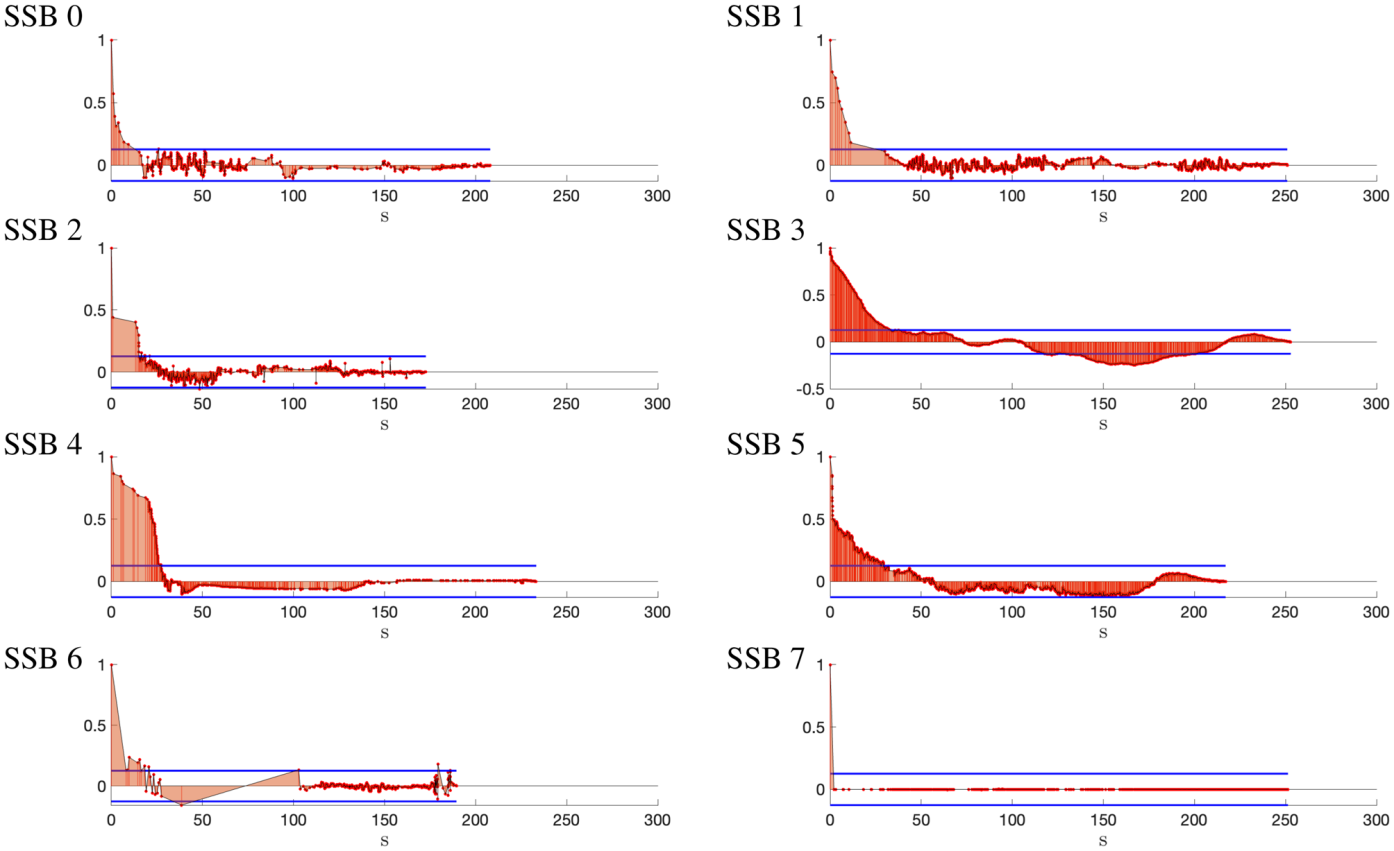
Normalized autocorrelation function of the range biases of TRP with PCI 252 for each SSBs.

### Clock offset coherence time

The second analysis focuses on the temporal variability of the synchronization error, with the objective of quantifying the coherence time defined as the time over which the clock error auto-correlation coefficient drops below a predefined threshold. This is extremely useful to understand how often information on clock correction should be provided to the UEs (in case of UE-based localization). The normalized autocorrelation function^37^ has been computed and compared to the threshold obtained using two standard errors of the sample autocorrelation as the confidence bounds. We refer to the Section Methods for more details. With this purpose, we consider the PCI 252 (which is in visibility) and plot in Fig. 6 the (normalized) autocorrelation function of the range error for each SSB beam. From the plots in the figure, we first notice that the range error of SSB 3 is more correlated over time (i.e., more stable over time), compared to the other SSBs. This behavior was expected as the beam for SSB 3 is directed towards the location of the scanner, i.e., along the geometrical LOS. On the contrary, the range error extracted from other beams have shorter coherence time (e.g., the autocorrelation is even impulsive for SSB 7), demonstrating the uncorrelation property of a range bias which is frequently subject to multipath phenomenon. Multipath main effects are random spikes in the range bias, which increase the signal decorrelation. It follows that the analysis on the autocorrelation properties can be used to detect the LOS beam. This is verified by the analysis on the received power in Section Methods where we clearly identify SSB 3 as the beam along the direct path. As concerns the coherence time, by setting a threshold of 0.2 to the autocorrelation function, we get a coherence time of $30-35$ seconds. This value is indicative to suggest an update of clock synchronization with a period not larger than $30-35$ seconds. Note that after 75 seconds, even if in visibility, the range bias becomes completely uncorrelated since the autocorrelation drops below the confidence bounds ($\pm 2\sigma$), which are computed considering the range bias as a Gaussian white noise process with a standard deviation $\sigma$ of about $1/\sqrt{N}$, with *N* the number of range bias samples.

To complete the analysis, in Table  2, we show the coherence time, indicated with $T_{\text {COH}}$, and the associated mean power for each PCI (i.e., 252, 411 and 383) and SSB. Note that PCI 411 holds a similar behavior with respect to PCI 252 and that the SSB with higher coherence time corresponds to the higher received mean power, verifying the detection of LOS beams through the autocorrelation property.

**Table 2:** Clock offset coherence time $T_{\text {COH}}$ (Table 2a) and mean received power (Table 2b) per PCI for each SSB.

(a)									
		SSB index							
	$T_{\text {COH}}$ [s]	0	1	2	3	4	5	6	7
PCI	252	15.2	30.0	15.0	**33.3**	27.2	27.7	16.3	2.0
	411	24.2	0.8	**34.9**	22.4	0.6	NA	10.7	1.5
	383	33.3	62.9	84.3	0.2	**87.0**	81.9	81.6	0.3

(b)									
		SSB index							
	Mean power [dBm]	0	1	2	3	4	5	6	7
PCI	252	−104.8	−107.3	−107.3	**−91.1**	−102.5	−100.9	−108.5	−106.3
	411	−97.3	−97.7	**−87.0**	−91.3	−102.9	NA	−93.4	−94.3
	383	−93.3	−89.8	−86.5	−81.7	**−69.8**	−82.0	−86.8	−83.6

The SSBs with higher coherence time are highlighted in bold.

**Figure 7:**
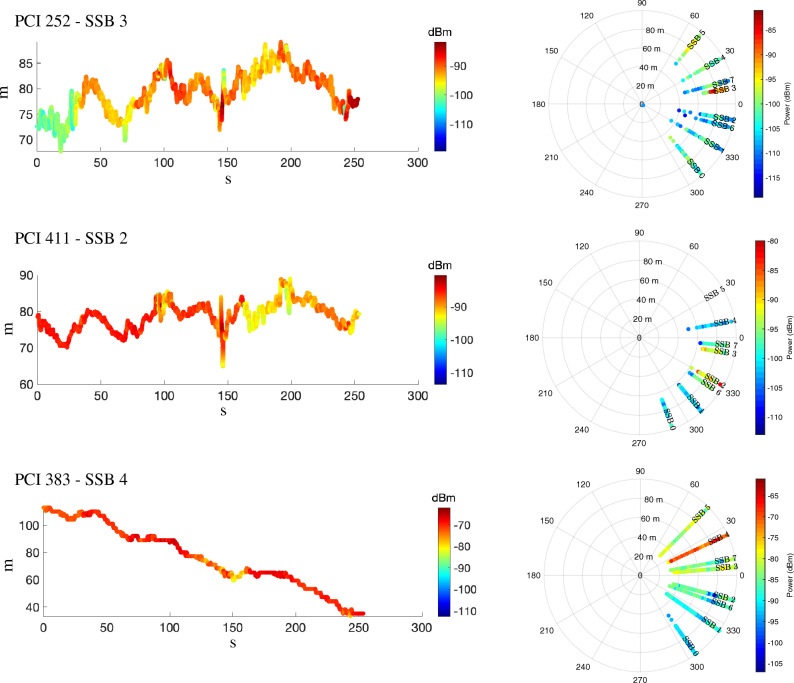
Evolution of range bias (in m) over time for PCI 252, 411 and 383 with corresponding 2D representation of received power per beam. Colors indicate the value of measured received power in dBm.

On the contrary, while the TRPs with PCI 252 and 411 hold a much narrow range bias distribution, the TRP with PCI 383 has a much higher drift in time. To highlight this aspect, in Fig. 7 we report the range biases, and associated received power, over the measuring time. To identify the LOS beam, we also report the corresponding power per SSB together with the associated range bias values in the radial axes. Considering the LOS beam of PCI 383, i.e., SSB 4, we measure a rate of change in range bias over time, i.e., clock drift, of 0.3 m/s which causes the wide support in Fig. 4. This is compatible with the OCXOs which are typically adopted in cellular TRPs^30^ and hold a drift between 0.1 and 10 part per billion (ppb), equivalent to 0.03 m/s and 3 m/s. In order to discuss the idea that the drift was caused by the near site distance, we point out that the drift was observed in all the three PCIs belonging to the site in discussion, i.e., PCI 382, 383 and 402, and in all five reception points. Given the severe observed offset, compensating for these types of errors becomes of paramount importance. Moreover, while stable range biases, as the ones belonging to PCI 252 and 411, can be compensated with an a-priori clock-offset estimation, the drift has to be constantly tracked and communicated as assistance data to the UEs.

### Positioning performance

This last assessment has the objective of verifying the impact of synchronization errors on the positioning performance of current 5G network deployments. In particular, we perform two experiments to verify the effect of estimating and compensating the range bias in UE localization.

**Figure 8:**
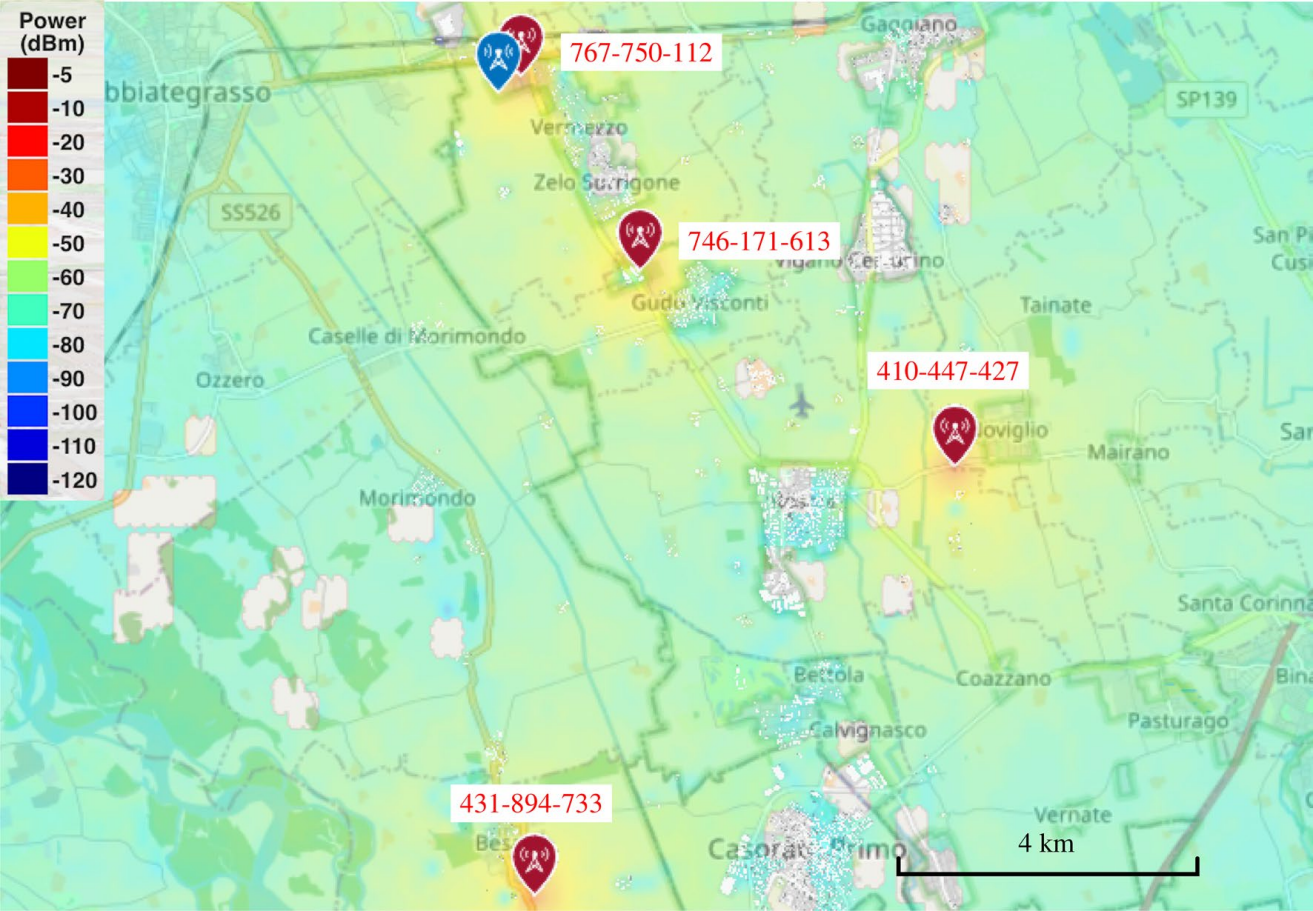
Simulated representation of the rural scenario composed of 12 TRPs (red icons) identified by the indicated PCI number and a static measurement point (blue icon). The power map is computed with MATLAB ray-tracing software at ground level.

We consider two different positioning scenarios, an urban area in Milan (Fig. 2) in the Leonardo Campus of the Politecnico di Milano and a rural area in the suburbs of Milan (see Fig. 8). In the former, the multipath is expected to be relevant and play a crucial role, often creating multi-modal distributions of range errors. In the latter, on the other hand, LOS condition is mostly available, but the larger distance between the TRPs and UE might cause a reduction of received power. For the evaluation of 5G positioning in the urban scenario, we considered three TRPs in LOS, i.e., PCIs 383, 411 and 252, while for the rural scenario we selected four TRPs in LOS, i.e., PCIs 733, 427, 613 and 750. Regarding the synchronization technology, PCI 733 adopts the Precision Time (PTPv2) protocol, while PCIs 427, 613 and 750 use SFP via GNSS. The selection of LOS TRPs is based on both the analysis of clock offset distributions, as described in Section Clock offset statistics, and from geographical positions with respect to the UE.

Three different localization methods are compared: a snapshot (i.e., without tracking over time) Non-linear Least Squares (NLS) algorithm without range bias correction (blue scatter points); an NLS with range bias correction (green scatter points); a tracking over time by an Extended Kalman Filter (EKF) with range bias correction (purple scatter points). For each method, the 95% confidence ellipse (95% of estimates fall inside the ellipse) is reported. The CramérRao Bound (CRB) ellipse (in yellow), considering the standard deviation on the range bias and achievable accuracy with the given bandwidth, is shown as reference. Lastly, the blue icon indicates the UE ground truth position; while the red icons identify the TRPs. The mean of the position estimate and the error bias are denoted with a black star marker and solid black lines, respectively. For the assessment, we considered the following performance metrics: Mean Absolute Error (MAE), Distance Root Mean Square (DRMS), and error ellipse with 95% confidence. Moreover, for each method, we plot the Cumulative Density Function (CDF) of UE positioning error, while a complete list of results, i.e., MAE, Circular Error Probable (CEP) 95, bias norm and DRMS, for both urban and rural scenarios, is reported in Table 3.

**Table 3:** UE positioning: performance comparison.

Method	Metric	Urban	Rural
NLS without range bias correction	DRMS [m]	67.35	35.20
	CEP 95 [m]	134.71	70.41
	MAE [m]	111.35	141.05
	Bias [m]	110.27	134.52
NLS with range bias correction	DRMS [m]	8.20	21.35
	CEP 95 [m]	16.41	42.71
	MAE [m]	10.79	24.60
	Bias [m]	3.66	11.84
EKF with range bias correction	DRMS [m]	3.93	7.62
	CEP 95 [m]	7.87	15.25
	MAE [m]	8.22	10.70
	Bias [m]	2.38	9.69
Empirical CRB	DRMS [m]	5.18	2.93
	CEP 95 [m]	10.36	5.86
Theoretical CRB	DRMS [m]	3.99	1.68
	CEP 95 [m]	7.98	3.36

**Figure 9:**
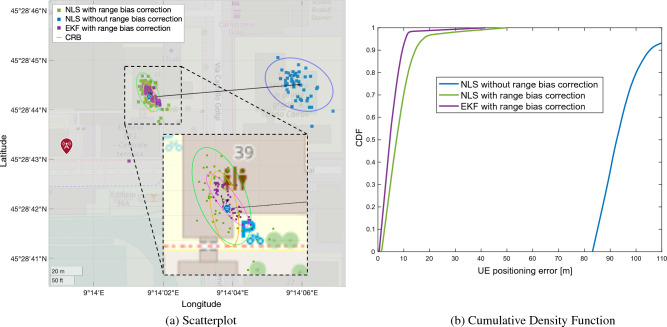
Urban scenario: (**a**) scatterplot of the position estimates in case of range correction (green squares), without range correction (blue squares) and EKF. The ellipses are obtained with 95% of confidence level. Black squares and solid black lines represent the mean position estimates and the error bias, respectively, while the CRB is highlighted with orange line. (**b**) CDF of the UE positioning error.

**Figure 10:**
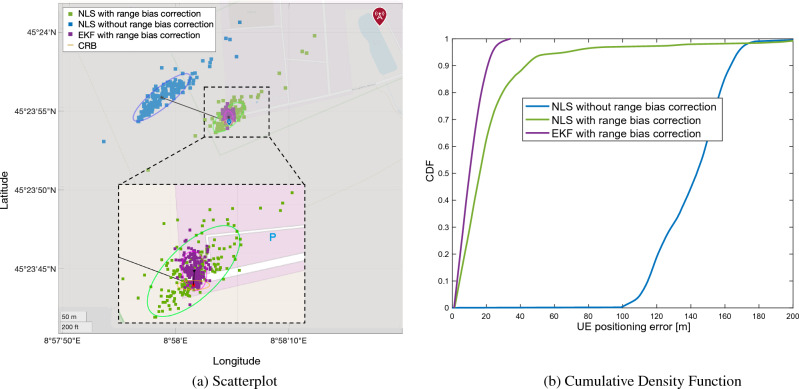
Rural scenario: (**a**) scatterplot of the positions estimates in case of range correction (green squares), without range correction (blue squares) and with EKF. The ellipses are obtained with 95% of confidence level. Black squares and solid black lines represent the mean position estimates and the error bias, respectively, while the CRB is highlighted with orange line. (**b**) CDF of the UE positioning error.

The results of static UE positioning for the urban scenario are presented in Fig. 9. Comparing the NLS approaches, we remark the beneficial effect of range bias correction, which reduces the MAE of UE localization from 111 m to 10 m. Analyzing the variance of UE location estimates (removing the bias) we can use the CRB as benchmark. Specifically, the NLS with range bias correction has a CEP 95 of 16.4 m which is higher than the CRB of 10.3 m. Adopting a tracking filter for UE positioning and by taking into account the correlation of the UE position over time, a further performance improvement over NLS is achieved: including mobility information of quasi-static UE, the EKF achieves an MAE of 8.2 m, with CEP 95 of 7.8 m.

Moving to the rural scenario, we have an increased number of TRPs in visibility but with a much higher distance compared to the urban area (see the scale in the bottom-right corner of Figs. 2 and 8). The results of the analyses are reported in Fig. 10, where the same methods adopted in the urban area are used. The UE localization with NLS without range bias correction achieves an MAE of 141 m, i.e., 30 m bigger than the urban case. The range correction and use of EKF lead to increased positioning performance, with MAEs of 24.6 m and 10.7 m, respectively.

### Discussion

According to the analyses and results reported in Section Results, we can conclude that synchronization constitutes a major issue for precise cellular positioning. Considering the current network deployments characterized by 3GPP Release 15 standard, relative-narrow bandwidth of SSBs (7.2 MHz) and high clock drifts between TRPs, a positioning accuracy of 8.2 m and 10.7 m has been achieved in urban and rural environments, respectively, upon compensating for synchronization errors. We remark that this is done without any alteration of the network and gathering only passive signals. To achieve such performance, a tracking filter together with almost perfect correction of synchronization errors has been used.

While the deployment of future 5G releases should improve cellular coverage and use larger signal bandwidth (e.g., by the PRSs in 3GPP Release 16), network synchronization will still remain a great challenge. In contexts where the relative time error between nearby base stations is the most important factor, novel synchronization algorithms are being defined. Promising solutions are the over-the-air synchronization (OAS)^38^ or even the optical fiber synchronization^39^.

As explained in Section Methods, we adopted a single 5G scanner for the experiments, gathering separately, i.e., at different time, the range bias statistics and the measurements adopted for positioning. This led to an unavoidable positioning error quantified with the standard deviation of the range-biases distributions ($13-15$ m in our case). In order to obtain cm-level accuracy, as required for challenging applications (e.g., cellular positioning for automated driving^40, 41^), range bias should be measured and shared with the UEs.

Future works could extend our study in this direction, exploiting not only higher signal bandwidths, but also precise beam management and joint use of multi-constellation GNSS and 5G observations. Moreover, advanced synchronization techniques to track in real-time the range corrections are also of interest, as well as the use of filtering techniques to jointly estimate the UE position and clock offsets of TRPs.

## Methods

To study the effect of synchronization in the currently deployed 5G networks, we conducted a number of experiments with a R&S TSMA6 scanner^42^, which is capable of simultaneously measuring signals between 350 MHz and 6 GHz. The TSMA6 scanner makes use of an internal pulse per second (PPS) signal generator to determine the time of arrival of a 5G signal. The scanner requires a configuration procedure to select the frequency bands, load modules (e.g., 5G scanner and GNSS), and acquire GNSS signal. When it is fully configured, it is able to simultaneously measure multiple 5G signals (from different TRPs), outputting a measurement every 20 ms (the sampling frequency of the instrument is 30.72 MHz). Among them, the ones of interest for our study are the SSBs. Specifically, the scanner outputs the ToA of each detected SSB: this information is available under the technical name of “ToA PPS”^42^ since it uses the GNSS PPS for time reference. The “ToA PPS” refers to the time of arrival of the PSS within the SSB. We shall notice that a post-processing is performed by the scanner in order to retrieve the final Time of Arrival (ToA) value. The scanner’s internal processing involves the computation of the cross-correlation of the received signals, which is used to determine the top-*N* multipath components (i.e., the *N* components with the highest cross-correlation value). The decision value for this ranking is set to the average Signal-to- Interference-plus-Noise Ratio (SINR), which provides a balance between signal strength and quality. Among these top-*N* multipath components, the one with the lowest “ToA PPS” is selected for ToA estimation. This approach ensures robustness in mitigating the ambiguities due to early arrivals. It is important to note that the R&S scanner does not permit full access to physical level parameters, as it automatically performs TOA estimation processing. Therefore, the analyses we carried out are based on the processed output provided by the scanner. The datasheet of the R&S scanner specifies a granularity of 5 ns with good GNSS visibility. This level of precision allows for accurate measurements of the ToA of 5G signals, which is crucial for our study.

For measurement uncertainties, the scanner reports the chosen average SINR, which can be used as quality metrics of the measurements, and also the Synchronization Signal (SS)-Received Signal Received Power (RSRP)^7^, which we referred to as received power.

**Figure 11:**
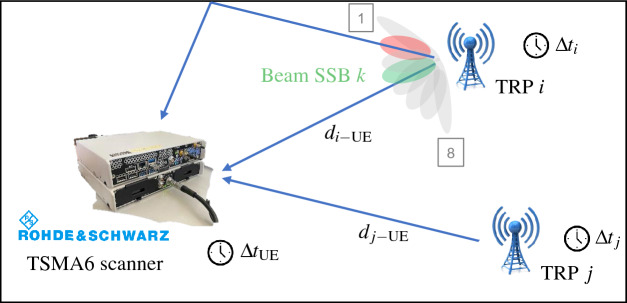
SSB beam sweeping along different directions. The receiver, i.e., R&S TSMA6 scanner, gathers both direct and reflected signals, experimenting a multipath delay. $\Delta t_i$ and $\Delta t_{\text {UE}}$ are the clock offset of the TRP *i* and UE, respectively. On the contrary, the distance between the TRP *i* and UE are indicated with $d_{i-\text {UE}}$.

In Fig. 11, we show a concise representation of beams, i.e., SSBs, sent by the TRPs. Since the SSBs are transmitted over different spatial directions, the scanner acquires signals coming from both LOS and NLOS beams. Knowing the 5G frame structure, the employed numerology, and SSB transmission pattern and measuring the ToA of each SSB (if any), we estimate the distance between the transmitting TRP and receiving UE (i.e., the scanner). The measured delay of the received signal is composed of the Time of Flight (ToF) of the propagation, multipath effects, the additional contribution of desynchronization, and other sources of unknown errors inside the hardware.

We define with $t_{\text {tx}}^{\text {(Start)}}$ the time of transmission of the beginning of the 5G frame at the TRP, with $t_{\text {tx}, i}^{(k)}$ and $t_{\text {rx}, i}^{(k)}$ the instants of transmission of the SSB *k* at TRP *i* and its reception at the scanner, respectively. The clock offsets of the receiver (i.e., the scanner) and of TRP *i* are indicated with $\Delta t_{\text {UE}}$ and $\Delta t_i$, respectively. Defining with $\textbf{p}^{\text {TRP}}_i$ and $\textbf{x}$ the position of the TRP *i* and of the scanner, respectively, our goal is to estimate the true ToF $\tau _{i}^{(k)} = t_{\text {rx}, i}^{(k)} - t_{\text {tx}, i}^{(k)}$ from TRP *i* and SSB *k* to the scanner. The estimate is defined as:

1 $$\begin{aligned} \widehat{\tau }_{i}^{(k)} = t_{\text {rx}, i}^{(k)} + \Delta t_i - \left( t_{\text {tx}, i}^{(k)} + \Delta t_{\text {UE}}\right) + {\Delta t}_{\text {mp}} + {\Delta t}_{\text {n}} \approx \tau _{i}^{(k)} + \Delta t_i - \Delta t_{\text {UE}}, \end{aligned}$$

where ${\Delta t}_{\text {mp}}$ and ${\Delta t}_{\text {n}}$ are the range errors due to multipath and noise at the radio receiver, respectively. Given that our experiments were conducted in controlled (to the best of our possibilities) LOS conditions, where multipath effects are minimized and LOS is mainly guaranteed (at least for one beam), we assume the ToA measurements gathered by the R&S equipment refer to the direct path. As concerns the receiver noise, we observe that the standard deviation of the ranging error due to this source of error is nearly an order of magnitude smaller than the standard deviation of synchronization error. For this reason, we focus on the range biases due to synchronization error only, neglecting the characterization of error contributions ${\Delta t}_{\text {mp}}$ and ${\Delta t}_{\text {n}}$, leading to the approximation in (1).

The ToA measured by the receiving scanner for TRP *i* and SSB *k* is:

2 $$\begin{aligned} \widehat{\text {ToA}}_{i}^{(k)} &= t_{\text {rx}, i}^{(k)} - t_{\text {tx}}^{\text {(Start)}} + \Delta t_i - \Delta t_{\text {UE}} +{\Delta t}_{\text {mp}} + {\Delta t}_{\text {n}} \\ &\approx t_{\text {rx}, i}^{(k)} - t_{\text {tx}}^{\text {(Start)}} + \Delta t_i - \Delta t_{\text {UE}}, \end{aligned}$$

thus it is possible to compute the ToF by subtracting the time interval between the start of the 5G frame $t_{\text {tx}}^{\text {(Start)}}$ and the expected transmission of the SSB $t_{\text {tx}, i}^{(k)}$ to the measured arrival time $\widehat{\text {ToA}}_{i}^{(k)}$. As an example, the interval $t_{\text {tx}, i}^{(0)} - t_{\text {tx}}^{\text {(Start)}}$ for SSB 0 with numerology 1, $f=3.68$ GHz and SSB Case C (see Fig. 1) is about 2 OFDM symbol intervals, i.e., 71.875 $\mu$s. We can then transform (1) into:

3 $$\begin{aligned} \widehat{\tau }_{i}^{(k)} &= \widehat{\text {ToA}}_{i}^{(k)} - \left( t_{\text {tx}, i}^{(k)} - t_{\text {tx}}^{\text {(Start)}} \right) \\ &\approx \left( t_{\text {rx}, i}^{(k)} - t_{\text {tx}}^{\text {(Start)}} + \Delta t_i - \Delta t_{\text {UE}}\right) - \left( t_{\text {tx}, i}^{(k)} - t_{\text {tx}}^{\text {(Start)}}\right) . \end{aligned}$$

This is possible if both the R&S and the TRPs have the common reference GNSS time and, therefore, they are able to nominally compute the same start of the frame $t_{\text {tx}}^{\text {(Start)}}$ (except from the local clock errors).

After having measured the ToF with (3), by knowing the exact distance between the transmitting TRPs and the receiving UE it is possible to compute the synchronization error $e^{(k)}_{synch,i}$ between TRP *i* and UE as:

4 $$\begin{aligned} e^{(k)}_{synch,i} &= \widehat{\tau }_{i}^{(k)} - \left( \frac{d_{i-\text {UE}}}{c}\right) \approx t_{\text {rx}, i}^{(k)} + \Delta t_i - t_{\text {tx}, i}^{(k)} - \Delta t_{\text {UE}} - \left( t_{\text {rx}, i}^{(k)} - t_{\text {tx}, i}^{(k)}\right) \\ &= \Delta t_i - \Delta t_{\text {UE}} , \end{aligned}$$

where $d_{i-\text {UE}} = \Vert \textbf{p}^{\text {TRP}}_i- \textbf{x} \Vert _{2}$ is the distance between TRP *i* and the receiving scanner, while *c* is the propagation speed. The positions of the measurement points have been measured with Real-Time Kinematic (RTK) technology, and positioning services provided by SPIN3 GNSS^43^, by placing the RTK receiver right below the 5G antenna of the R&S scanner. Since RTK technology guarantees an accuracy of few centimeters^44^, considering the final error on 5G positioning in the order of several meters, we can safely use the RTK output as the exact UE coordinate. On the other hand, the coordinates of TRPs were measured by merging RTK surveys of the site boundaries (we remark that accessing the 5G sites and reaching the heights of the antennas is extremely challenging) with architectural drawings of the sites, resulting into highly-reliable information in the order of decimeters and still much lower than the 5G positioning error. We highlight that the clock difference $\Delta t_i - \Delta t_{\text {UE}}$ cannot be separated into the two offset components as they are related to perfectly synchronized time-axes which cannot be measured. Therefore, we can just measure the difference in offset between two asynchronous clocks.

**Figure 12:**
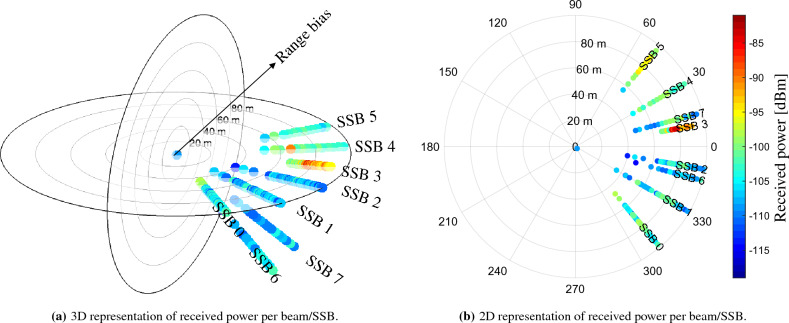
The radial axes represents the range bias in meters while the color indicates the received power for each measure. (**a**) and (**b**) show the beams in 3D and 2D, respectively.

The ranging error will only consist of the offset between the clock of the scanner and the clock of the TRPs, under the assumption of LOS propagation. To guarantee such assumption, we need to understand which beam is in LOS, i.e., which beam is pointing towards the scanner. To this aim, knowing the azimuth and elevation of the panel array of the TRP, in Fig. 12 we report the associated power per SSB, i.e., per beam, together with the associated range bias values in the radial axes. By knowing the technical implementation features of the Vodafone 5G TRPs, we are able to state that the represented TRP in LOS (PCI 252) covers a spatial sector of 120 degrees in azimuth with 6 high-beams of roughly 20 deg beamwidth each and 2 low-beams of roughly 60 deg beamwidth each. The high-beams are referred to SSB numbers 0, 1, 2, 3, 4 and 5, while the low-beams with SSB numbers 6 and 7. For this specific TRP with PCI 252 in visibility condition, the beam with higher received power is the SSB number 3, with an average received power of about $-90$ dBm. On the contrary, the received power for the other beams is as low as $-120$ dBm. A peculiar fact is that, analyzing the spreads of the range bias samples, they appear to hold the same support, i.e., about 60 m as also shown in Fig. 4. This is due to the fact that the scanner is able to receive even very attenuated signals and that the low-power received signals are obtained from a direct path. Indeed, the radiation pattern of the antenna panel, even if highly directional, does not retain a null gain in the undesired directions of propagation. This aspect can be non-optimal for communication aspects (where high received power enables higher throughput), but it is still useful for positioning (where the key information is about the shortest path).

For the study in Section Clock offset coherence time, we computed the normalized autocorrelation function of the clock error time series $y_t$ as^37^:

5 $$\begin{aligned} r_k = \frac{\sum _{t=1}^{T-k} (y_t - \bar{y})(y_{t+k} - \bar{y})}{\sum _{t=1}^T (y_t - \bar{y})^2} , \end{aligned}$$

where $r_k$ is the autocorrelation at lag *k*, $\bar{y}$ is the mean of the time series and *T* is the total number of observations. The coherence time is defined as the lag over which the autocorrelation drops below a given threshold. The threshold is obtained using two standard errors of the sample autocorrelation in the confidence bounds, where the formula for the standard error using Bartlett’s approximation is:

6 $$\begin{aligned} SE = \sqrt{\frac{1}{T}\left( 1 + 2\sum _{i=1}^{q} r_i^2\right) } \, , \end{aligned}$$

where *q* is the lag beyond which the autocorrelation function is 0.

Given the availability of a single 5G scanner, we carried out the range bias estimation and measurement collection for UE positioning on two separate datasets. In the urban scenario, we considered four reception points for range error computation at the beginning of the experiment and one reception point at the end for testing. On the contrary, in the rural area, we did the opposite procedure. Both the testing and measurements phases last about 250 s, while the intervals between each measurement point in both scenarios were approximately of 10 minutes. Given the unfeasibility of tracking in real-time the range biases, we used the median values of each range bias distribution as a-priori compensation, which were then averaged across the measurement points.

We should also note that, for real applications, the computed coherence time should serve as a reference time interval for estimating the clock offsets. In other words, range biases should be estimated and compensated at least once within the coherence time, or even more such as by continuously tracking.

UE positioning is performed every *T* seconds by gathering all ToF measurements from all the measured SSBs within the temporal window. Denoting with $\varvec{\widehat{\tau }}_{i} = \{\widehat{\tau }_{i}(\ell )\}_{\ell =1}^{L_i}$ the set of ToF measurements from TRP measurements from TRP *i* at position $\textbf{p}^{\text {TRP}}_i$ in the interval *T*, we compute the median value over the measured ToF for each TRP such that the aggregated vector $\varvec{\rho }$ of all ToF measurements is created as:

7 $$\begin{aligned} \varvec{\rho } = \begin{bmatrix} \rho _{1} \\ \vdots \\ \rho _{N} \end{bmatrix} = \begin{bmatrix} \text {med}(\varvec{\widehat{\tau }}_{1}) \\ \vdots \\ \text {med}(\varvec{\widehat{\tau }}_{N}) \end{bmatrix}, \end{aligned}$$

where $\text {med}(\cdot )$ is the median operator and *N* is the number of detected TRPs. Then, the UE bidimensional position estimate $\widehat{\textbf{x}}$ , is computed according to the NLS algorithm with Jacobian matrix $[\textbf{H}]_{i-\text {th row}} = [\textbf{H}(\textbf{x})]_{i-\text {th row}} = \frac{\partial \text {h}_i(\textbf{x})}{\partial \textbf{x}}$, where $\text {h}_i(\textbf{x}) =\Vert \textbf{p}^{\text {TRP}}_i- \textbf{x} \Vert _{2}$ is the ToF measurement model. The NLS algorithm is implemented with the iterative search of the estimate according to the Gauss-Newton methodology^45^ or EKF^46^ with random walk motion model^47^.

The 95% confidence ellipse are obtained from the error covariance matrix $\textbf{C}$ defined as:

8 $$\begin{aligned} \textbf{C} = \mathbb {E}[\Delta \widehat{\textbf{x}} \Delta \widehat{\textbf{x}}^{\text {T}}]\,, \end{aligned}$$

where $\Delta \widehat{\textbf{x}} = \widehat{\textbf{x}} - \mathbb {E}[\widehat{\textbf{x}}]$. The 95% confidence level is computed considering an horizontal accuracy of $2\sigma _H = 2\sqrt{{{\,\textrm{Tr}\,}}{(\textbf{C})}}$, where ${{\,\textrm{Tr}\,}}{(\cdot )}$ denotes the trace of the argument matrix. Finally, the MAE can be extracted as $\text {MAE} = \mathbb {E}[\Vert \widehat{\textbf{x}}- \textbf{x} \Vert _{2}]$.

In order to have a lower bound on the positioning error, we computed the CRB by deriving the Fisher information matrix of the range-based localization problem^48^, which accounts for both the TOF measurement error and the geometrical condition of TRPs and UE. For CRB computation, we assumed the TOF measurements as Gaussian, unbiased and uncorrelated, and each link as single-path LOS. It follows that the standard deviation of the ranging measurement of TRP *i* is:

9 $$\begin{aligned} \sigma _{\tau , i} = \frac{c}{2\sqrt{2} \pi \sqrt{SNR_{i}} \,\beta }\,, \end{aligned}$$

where $\beta$ is the effective bandwidth (here assumed to be equal to 7.2 MHz), and $SNR_{i} \triangleq E_{s, i} /N_0$ is the signal-to-noise ratio which is directly computed by the R&S scanner. $E_{s,i}$ is the average received energy from TRP *i*, while $N_0/2$ represents the spectral density of the additive white Gaussian noise.

The final CRB covariance matrix $\textbf{C}_{\text {CRB}}$ of the position estimate is obtained as:

10 $$\begin{aligned} \textbf{C}_{\text {CRB}} = \sigma _{\tau }^{2} \textbf{G}\,, \end{aligned}$$

where $\textbf{G} = (\textbf{H}^{\text {T}}\textbf{H})^{-1}$ is the Geometric Dilution of Precision (GDoP)^49^ of the positioning problem and $\sigma _{\tau } = \sqrt{{\mathbb {E}}_{{i}}\Big \{{\sigma _{\tau , i}^2}\Big \}}$.

## Data Availability

The datasets generated and analyzed during the current study are not publicly available due restrictions imposed by the European Space Agency, who funded this work under NAVISP Programmes, Activity Code: NAVISP-EL2-114, but are available from the corresponding author on reasonable request and under a Non-Disclosure Agreement.
