# Author Correction: A feasibility study of 5G positioning with current cellular network deployment

**DOI:** 10.1038/s41598-023-43190-y

**Published:** 2023-09-25

**Authors:** Bernardo Camajori Tedeschini, Mattia Brambilla, Lorenzo Italiano, Simone Reggiani, Davide Vaccarono, Marianna Alghisi, Lorenzo Benvenuto, Alessandro Goia, Eugenio Realini, Florin Grec, Monica Nicoli

**Affiliations:** 1 Dipartimento di Elettronica, Informazione e Bioingegneria (DEIB), Politecnico di Milano, 20133 Milan, Italy; 2 Network Engineering, NTT DATA, 20143 Milan, Italy; 3 Network Strategy and Engineering, Vodafone Italia, 10155 Ivrea, Italy; 4 Dipartimento di Ingegneria Civile e Ambientale (DICA), Politecnico di Milano, 20133 Milan, Italy; 5 algoWatt S.p.A., 16149 Genoa, Italy; 6 Geomatics Research & Development srl (GReD), 22074 Lomazzo, Italy; 7 European Space Agency (ESA), 2201 AZ Noordwijk, The Netherlands; 8 Dipartimento di Ingegneria Gestionale (DIG), Politecnico di Milano, 20133 Milan, Italy

Correction to: *Scientific Reports* 10.1038/s41598-023-42426-1, published online 15 September 2023

The original version of this Article contained errors in affiliations of Lorenzo Benvenuto and Eugenio Realini. Their affiliations were inadvertently switched.

The correct affiliation for Lorenzo Benvenuto is listed below:

‘algoWatt S.p.A., 16149 Genoa, Italy.’

The correct affiliation for Eugenio Realini is listed below:

‘Geomatics Research & Development srl (GReD), 22074 Lomazzo, Italy’.

The original Article has been corrected.

